# Resveratrol, Acetyl-Resveratrol, and Polydatin Exhibit Antigrowth Activity against 3D Cell Aggregates of the SKOV-3 and OVCAR-8 Ovarian Cancer Cell Lines

**DOI:** 10.1155/2015/279591

**Published:** 2015-11-05

**Authors:** Simon J. Hogg, Kenny Chitcholtan, Wafaa Hassan, Peter H. Sykes, Ashley Garrill

**Affiliations:** ^1^Peter MacCallum Cancer Centre, St Andrews Place, East Melbourne, Melbourne, VIC 3002, Australia; ^2^Department of Obstetrics and Gynaecology, University of Otago, Christchurch, 2 Riccarton Avenue, Christchurch 8011, New Zealand; ^3^School of Biological Sciences, University of Canterbury, Private Bag 4800, Christchurch 8140, New Zealand

## Abstract

Resveratrol has aroused significant scientific interest as it has been claimed that it exhibits a spectrum of health benefits. These include effects as an anti-inflammatory and an antitumour compound. The purpose of this study was to investigate and compare any potential antigrowth effects of resveratrol and two of its derivatives, acetyl-resveratrol and polydatin, on 3D cell aggregates of the EGFR/Her-2 positive and negative ovarian cancer cell lines SKOV-3 and OVCAR-8, respectively. Results showed that resveratrol and acetyl-resveratrol reduced cell growth in the SKOV-3 and OVCAR-8 in a dose-dependant manner. The growth reduction was mediated by the induction of apoptosis via the cleavage of poly(ADP-ribose) polymerase (PARP-1). At lower concentrations, 5 and 10 *µ*M, resveratrol, acetyl-resveratrol, and polydatin were less effective than higher concentrations, 50 and 100 *µ*M. In SKOV-3 line, at higher concentrations, resveratrol and polydatin significantly reduced the phosphorylation of Her-2 and EGFR and the expression of Erk. Acetyl-resveratrol, on the other hand, did not change the activation of Her-2 and EGFR. Resveratrol, acetyl-resveratrol, and polydatin suppressed the secretion of VEGF in a dose-dependant fashion. In the OVCAR-8 cell line, resveratrol and acetyl-resveratrol at 5 and 10 *µ*M increased the activation of Erk. Above these concentrations they decreased activation. Polydatin did not produce this effect. This study demonstrates that resveratrol and its derivatives may inhibit growth of 3D cell aggregates of ovarian cancer cell lines via different signalling molecules. Resveratrol and its derivatives, therefore, warrant further* in vivo *evaluation to assess their potential clinical utility.

## 1. Introduction

Ovarian cancer is a lethal malignancy, and the prognosis is very poor for women who present with an advanced stage of the disease [1]. As chemotherapy is not normally curative in women with advanced ovarian cancers, treatments that can slow the growth of tumours and, hence, prolong the life of those with the disease are of great importance. Advanced ovarian cancer is often associated with ascites, the excess accumulation of the body fluid in the abdominal cavity [2, 3]. The permeability of blood and lymphatic vessels at the peritoneal wall is compromised by high levels of vascular endothelial growth factor (VEGF) and that causes ascites. Ascitic fluid which can move freely around the abdominal cavity contains malignant ovarian cells; these cells can become deposited on the surface of peritoneal membrane, thereby establishing multiple sites of secondary growth. The floating cancer cells often form small 3D aggregates, which may help them to survive as they circulate in the abdominal cavity [3]. As a result, these 3D cell aggregates may contribute to a likely source of cancer cells that have the ability to form small tumours and later grow in uncontrolled manner. Little is known about how ovarian cancer aggregates survive in ascitic fluid. Also, there have been limited studies that focus on the use of possible therapeutic targets in these ovarian cancer aggregates.

One possible chemotherapeutic target of ovarian cancers would be the inhibition of the tyrosine kinase receptors, ErBb1 (EGFR) and ErBb2 (Her-2). These receptors have been reported to play an important role in a subset of ovarian cancers. The elevated expression of these receptors is typically correlated with a poor prognosis, and thus targeting their activation could potentially affect tumour growth [4]. The activation of EGFR and Her-2 elicits a vast array of signalling proteins responsible for cell proliferation, survival, metastasis, invasion, ECM remodelling, and angiogenesis [5]. The MAPK activation pathway and the production of VEGF are affected by suppressing EGFR/Her-2 activation [5]. However, the role of EGFR and Her-2 in ovarian cancer aggregates is still poorly understood. A compound that has the ability to suppress the activation of these receptors may be of importance in controlling the progression of ovarian cancer aggregates and ascites.

**Figure 1:**
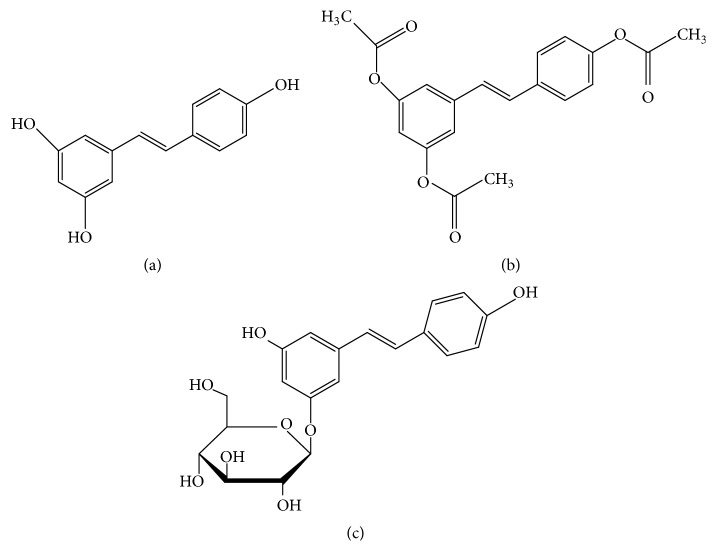
The chemical structures of resveratrol (a), acetyl-resveratrol (b), and polydatin (c).

Resveratrol (3,5,4′-trihydroxy-trans-stilbene) (Figure 1(a)) and its derivative compounds, acetyl-resveratrol (3,5,4′-tri-O-acetylresveratrol) (Figure 1(b)) and polydatin (3,5,4′-trihydroxy-stilbene-3-beta-mono-D-glucoside) (Figure 1(c)), are natural occurring polyphenol compounds that have aroused interest due to their potential health benefits. Resveratrol has been shown to affect the activation of EGFR and Her-2 in several types of cancers in preclinical studies [6]. Furthermore, EGFR/Her-2 associated signalling proteins in PI-3K and MAPK pathways have been reported to be affected and responsible for resveratrol's antitumour activities [6]. Despite promising antitumour effects of resveratrol in* in vitro* studies, its inhibition of tumour progression in animal models is less well defined and is quite variable. Some studies have documented the growth reduction of tumours after a duration of resveratrol ingestion, but others have not noticed any antitumour effects of resveratrol [7, 8]. Furthermore, resveratrol has also been shown to increase tumour growth in certain types of cancers [9]. The discrepancy of antitumour properties of resveratrol between* in vitro* and* in vivo* studies may be partly due to the bioavailability of active molecules of resveratrol.

The use of resveratrol as a potential anticancer drug in humans is still controversial [10]. One significant drawback with respect to the clinical use of resveratrol for cancer patients is its typically low bioavailability [11]. Given this, more data with respect to its therapeutic efficacy against specific cancer types are needed to address its potential as a monotherapy or as an adjuvant regimen. Because of the possibility of limited usefulness of resveratrol itself due to the low plasma concentrations, there is a need to investigate compounds that are related to resveratrol and that may have greater bioavailability after ingestion. Two possible candidate compounds are acetyl-resveratrol and polydatin, which are currently under investigation for their cancer preventative properties [12, 13]. Polydatin has shown antitumour activities in* in vitro* studies [14, 15]. Polydatin has previously been reported to have a greater anti-inflammatory effect than resveratrol in a study of colitis in rats [16]. In animal studies, after ingestion, polydatin is metabolized to resveratrol and glucuronidated resveratrol in the intestines and the liver, respectively [17]. Furthermore, a native polydatin has been shown to be present at significant levels in the plasma of a mouse model [18]. Acetyl-resveratrol is metabolized and converted back to resveratrol that can be detected in plasma of a mouse model [7].

Clearly with respect to ovarian cancer aggregates, there is a need for more studies of the antitumour activities and the possible mechanistic effects of these compounds. Given the above, in this study we investigated whether resveratrol, acetyl-resveratrol, and polydatin elicited antigrowth of 3D cell aggregates of the EGFR/Her-2 positive cell line SKOV-3 and the EGFR/Her-2 negative cell line OVCAR-8. In addition, we presented preliminary studies of the mechanistic looking at the compounds potential modulation via both dependence on and independence of the expression of EGFR/Her-2.

## 2. Materials and Methods

The human ovarian adenocarcinoma cell line, SKOV-3, used in this project was purchased from ATCC. This cell line has high expression of EGFR and Her-2, PTEN wild type, E-cadherin negative, null* P53*, KRAS wild type, and BRAF wild type [19]. OVCAR-8 was obtained from Dr. Judith McKenzie, Haematology Research Group, University of Otago, Christchurch, New Zealand. This cell line has low expression of EGFR and Her-2,* P53* mutation, and E-cadherin negative [19]. SKOV-3 cells were maintained in Knockout Dulbecco's Modified Eagle Medium (DMEM Catalogue number 10829, GIBCO, ThermoFisher Scientific, New Zealand). This high glucose base medium was supplemented with 10% fetal bovine serum (FBS) (GIBCO, ThermoFisher Scientific, New Zealand), PenStrep (GIBCO, ThermoFisher Scientific, New Zealand) at a working concentration of 100 units/mL penicillin and 100 *μ*g/mL streptomycin, 2 mM GlutaMAX (GIBCO, Invitrogen, New Zealand), and 2 *μ*g/mL fungizone (ThermoFisher Scientific, New Zealand). OVCAR-8 was maintained in similar media, but it contained 5% FBS and 5% serum replacement (ThermoFisher Scientific, New Zealand). The supplemented DMEM media are henceforth referred to as working media. SKOV-3 and OVCAR-8 cells in the respective working media were maintained at 37°C in a humidified 5% CO_2_ atmosphere incubator. Both cell lines were continuously maintained in a culture flask.

### 2.1. Generation of 3D Cell Aggregates

To prevent adhesion of cells to culture plates and to encourage the formation of 3D aggregates, 12-well culture plates were precoated with 24 mg/mL poly-HEMA (Sigma, New Zealand) prior to cell culturing (0.6 mL/well). Prior to coating, poly-HEMA in 95% ethanol at a concentration of 24 mg/mL was heated to approximately 70°C to ensure that the poly-HEMA was fully dissolved. The coated plates were left overnight at 37°C on an orbital shaker. Prior to cell culture, the coated plates were washed once with PBS at pH 7.4. The cell monolayer of the SKOV-3 and OVCAR-8 cell lines was incubated with 1x trypsin-EDTA for 20–30 minutes to detach the cells from the surface of the flask. Cells were counted with a haemocytometer to determine the concentration of cells in the cell suspension. Cells were then plated at a density of 150,000 cells/well and were incubated at 37°C in a humidified 5% CO_2_ atmosphere for 6 days during which time the cells became compact aggregates. Over this period the media were removed by aspiration and replaced with 1.5 mL of fresh working media every 2 days for six days.

### 2.2. Treatment with Resveratrol and Derivatives

Resveratrol, acetyl-resveratrol, and polydatin powders were kindly provided by Dr. Saurabh Shah, Biotivia (USA). Acetyl-resveratrol was dissolved in 100% DMSO; polydatin and resveratrol were dissolved in a 50% : 50% combination of PBS and DMSO. For all experiment, the final amount of DMSO in controls and compound treated samples was 0.5% (vol/vol). Fresh working media containing the relevant compounds (at concentrations of 5, 10, 50, and 100 *μ*M) were replaced every 2 days for a total of 6 days. Thus, at the endpoint of cell culturing cells had a total incubation time of 12 days: 6 days for 3D aggregate formation and 6 days for the respective treatments. At least four independent experiments were carried out and each individual experiment was carried out with three replicates.

### 2.3. Analysis of Cell Cluster Morphology

Following 6 days of incubation to generate the cell aggregates and 6 days of treatment with compounds, images were collected using a camera (Leica DFC310FX, Germany) attached to an inverted light microscope (Leica DMI6000, Germany) with a 10x objective lens in order to assess the SKOV-3 cell aggregate morphology.

### 2.4. Growth Determination Using the Crystal Violet Assay

Growth of the 3D cell aggregates was quantified indirectly using crystal violet dye staining. In brief, cell aggregates were harvested and incubated with 1x trypsin-EDTA for 20 minutes at 37°C. Cells were washed twice with PBS pH 7.4 and were incubated for a further 30 minutes at 37°C with 2 mg/mL crystal violet in 2% (v/v) ethanol in deionized water. The cells were then washed with deionized water to remove unbound crystal violet dye and were harvested by centrifugation at 1500 rpm for 5 minutes. The supernatant was removed, and the process was repeated 3–5 times until the supernatant was colourless. Cells were then lysed in 10% (w/v) sodium dodecyl sulphate (SDS) solution. The homogenous cell lysate was diluted 3-fold in deionized water and 200 *μ*L of the samples was loaded onto a 96-well plate. The optical density was determined at 560 nm (OD_560_) using a microplate reader (SpectraMax M5, Molecular Devices). Measurements for each sample were made in triplicate.

### 2.5. Cellular Metabolism Determination Using the Alamar Blue Dye Assay

On the 5th day of treatment with compounds, 100 *μ*L of Alamar blue dye (ThermoFisher Scientific, New Zealand) in 1 mL media was added to cell aggregates. Cell aggregates were further incubated with the dye for 20 hours and 200 *μ*L culture media from each well was transferred to a 96-well plate. The absorbance at 570 and 600 nm was measured using a microplate reader (SpectraMax M5, Molecular Devices).

### 2.6. Detection of Vascular Endothelial Growth Factor (VEGF)

After 6 days of respective treatments, the media of the control and treated cells were used to determine secreted VEGF. Analysis of VEGF secretion was carried out using a DuoSet Human VEGF ELISA Kit (R&D Systems, New Zealand). The optical density of each well was measured using a microplate reader (SpectraMax M5, Molecular Devices) at 450 nm. The amount of VEGF in each sample was determined by comparing the absorbance of each of the wells to that of a standard curve. SigmaPlot (Systat Software, San Jose, CA) was used to create a standard curve from which VEGF concentrations of cell media samples were extrapolated.

### 2.7. Immunoblotting Analysis

Cell clusters were harvested by centrifugation at 1500 rpm for 5 minutes, and the cell pellet was resuspended in cold RIPA buffer containing protease inhibitor cocktail tablets (Complete Mini, Roche, New Zealand). The cell lysates were left on ice for a further 30 minutes to complete total lysis. Sample buffer (0.2% (v/v) bromophenol blue, 25% (v/v) glycerol, 10% SDS in Tris-HCl, and pH 6.8) was added and protein lysates were boiled for 10 minutes. Prior to loading, the cell lysates were mixed and centrifuged at 12,000 rpm for 5 minutes. Protein lysate was loaded and separated by SDS-PAGE using a 5% stacking gel and a 12% separating gel. The SDS-PAGE was run at 120 volts using Tris-glycine running buffer. The SDS-PAGE markers used were MagicMark Western Protein Standard (Invitrogen, New Zealand) and Precision Plus Protein standard (Bio-Rad, Hercules, USA). Separated proteins were electroblotted onto nitrocellulose membranes (0.45 microns, Trans-Blot Nitrocellulose Membrane, Bio-Rad, Hercules, USA). The electroblot was run at 100 Volts for 60 minutes in cold Tris-glycine running buffer containing 10% v/v methanol. The membranes were blocked for 60 minutes with either 5% (w/v) nonfat skim milk (Pams brand, New World, New Zealand) or 1% (w/v) bovine serum albumin (ThermoFisher Scientific, New Zealand) made up in TBS-T buffer or with Pierce Protein-Free Blocking Buffer (Thermo Scientific, New Zealand). Antibodies were diluted from 1 : 500 to 1 : 1000 with the appropriate blocking solution. Membranes were incubated with primary antibodies overnight at 4°C. The membranes were washed with TBS-T buffer on an orbital shaker for 4 × 10 minutes and then incubated with secondary antibody on an orbital shaker for 90 minutes at room temperature. Membranes were subsequently washed a further four times with TBS-T. Antibody localisation was determined using a chemiluminescent detection kit (Amersham ECL Prime Western Blotting Detection Reagent Kit, GE Healthcare). The protein bands were visualized and a densitometry analysis was performed using Alliance 4.7, Unitec (Cambridge, UK). Cell lysates were collected from at least 3 separated cell culture experiments. Antibodies were purchased from Santa Cruz Biotechnology Inc. (Santa Cruz, CA, USA). The primary antibodies used in this study were anti-PARP (sc-25780), anti-GAPDH (sc-25778), anti-pHER2 (sc-12352-R), anti-EGFR (sc-03), anti-pEGFR (sc-101668), anti-ERK (sc-94), and anti-pERK1/2 (sc-7383). Anti-Her-2 was purchased from BD Biosciences (Auckland, New Zealand). The two secondary antibodies used in this study were bovine anti-rabbit IgG-HRP (sc-2385) and bovine anti-mouse IgG-HRP (sc-2380).

### 2.8. Statistical Analysis

Data were statistically analyzed (SigmaPlot 11) using Student's *t*-test and ANOVA with *p* < 0.05 was considered to indicate statistical significance. All data are presented as mean ± SEM. Each experiment was repeated at least four times.

## 3. Results

### 3.1. Effects of Resveratrol and Its Derivatives on Cell Growth

**Figure 2:**
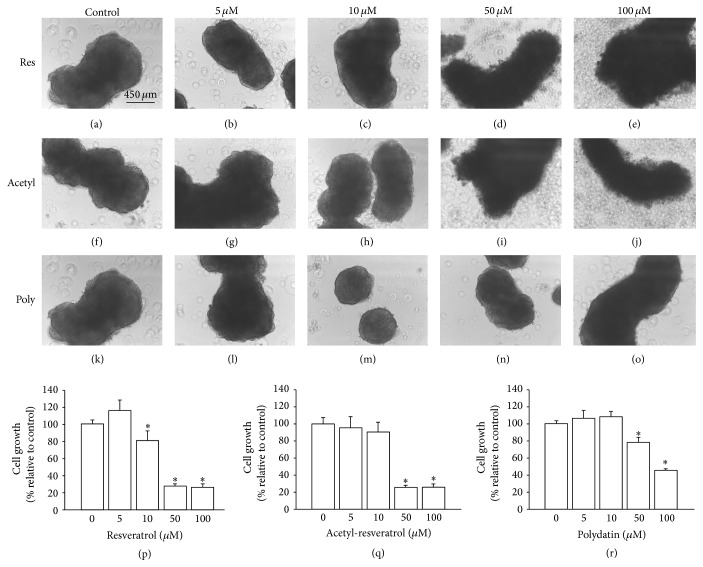
Morphological appearance of SKOV-3 cell aggregates with and without treatment. At higher concentrations all three compounds gave aggregates with irregular rims. The effect was more prominent with resveratrol (d, e) and acetyl-resveratrol (i, j) than with polydatin (n, o). The smooth rims of cell aggregates treated with lower concentration were not visibly different from those of control aggregates. Resveratrol significantly reduced cell growth at 10, 50, and 100 *μ*M (p), but acetyl-resveratrol (q) and polydatin (r) only affected growth activity at higher concentrations. *∗* refers to statistically significant as *p* value less than 0.05 compared to the control.

SKOV-3 cells formed large dense aggregates with circular, oval, and tubular structures in nontreated samples (Figures 2(a), 2(f), and 2(k)). This general morphology and aggregate size were not affected by the lower concentrations (5 *μ*M and 10 *μ*M) of all of the tested compounds. 3D aggregates of control, low concentration treated resveratrol (Figures 2(b) and 2(c)), acetyl-resveratrol (Figures 2(g) and 2(h)), and polydatin (Figures 2(l) and 2(m)) had a smooth rim. However, at the higher concentrations of resveratrol (Figures 2(d) and 2(e)) and acetyl-resveratrol (Figures 2(i) and 2(j)), aggregates had a rough irregular rim. Polydatin treated aggregates also showed an irregular rim, but this was less pronounced than in resveratrol and acetyl-resveratrol treated aggregates.

We further examined the relative growth of cell aggregates using a crystal violet assay. As shown in Figure 2(p), growth was reduced by about 70% with the higher concentrations (50 and 100 *μ*M) of resveratrol and by about 20% with the lower concentration of 10 *μ*M (Figure 2(p)). Similar growth reduction was also obtained with the higher concentrations of acetyl-resveratrol (Figure 2(q)). Polydatin showed less inhibition of growth. At concentrations of 50 and 100 *μ*M, it reduced growth by 20 and 50% (Figure 2(r)), respectively. There was no growth inhibition observed with the lower doses of acetyl-resveratrol and polydatin.

**Figure 3:**
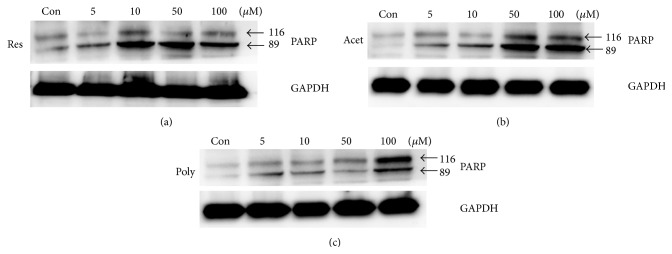
The effect of resveratrol (a), acetyl-resveratrol (b), and polydatin (c) on the expression of poly(ADP-ribose) polymerase (PARP) which was used as an indicator of apoptosis in SKOV-3 cell aggregates. At higher concentrations resveratrol, acetyl-resveratrol, and polydatin increased the fragmentation of PARP giving a band of 89 kDa which was indicative of apoptosis. At 10 *μ*M resveratrol induced the fragmentation of PARP. Based line expression of PARP at 116 and 89 kDa was observed in controls and low doses of all compounds.

To investigate whether apoptosis facilitating cell death caused the reduction of growth in 3D aggregate cells that were treated with the higher concentrations of the compounds, we examined the expression of the apoptotic marker protein, poly(ADP-ribose) polymerase (PARP). Resveratrol and acetyl-resveratrol increased the fragmentation of PARP giving a band with a molecular weight of 89 kDa; this is consistent with the fact that the reduction of cell number at the higher concentrations of compounds is due to apoptosis (Figures 3(a), 3(b), and 3(c)). The 89 kDa fragmented PARP was also observed in the control in addition to the treated samples, indicative of a baseline level of apoptosis in the cell clusters.

### 3.2. Effects of Resveratrol, Acetyl-Resveratrol, and Polydatin on Cell Signalling Molecules

**Figure 4:**
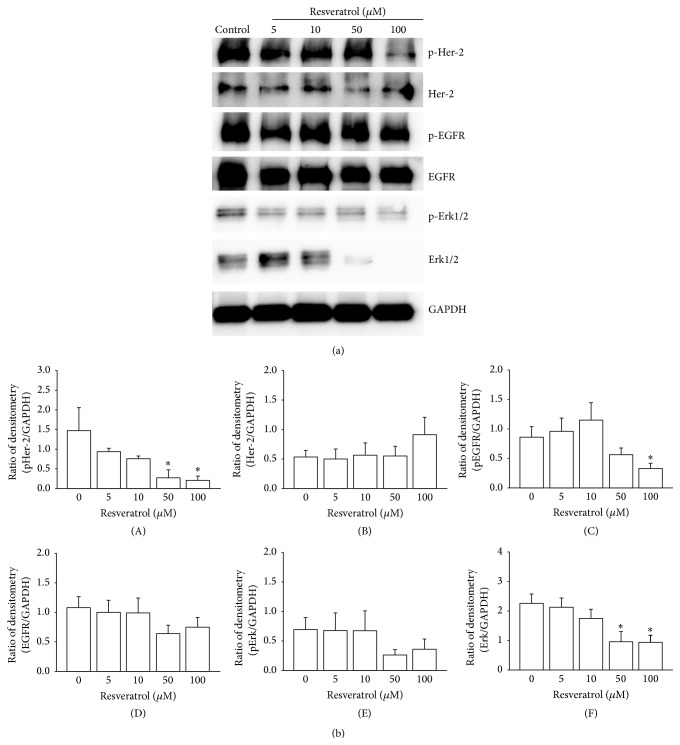
The expression and phosphorylation of Her-2, EGFR, Erk1/2, and GAPDH in SKOV-3 cell aggregates after treatment with resveratrol. Higher concentrations of resveratrol reduced the phosphorylation of Her-2 (A) and EGFR (C) and total expression of Erk1/2 (F). *∗* refers to statistically significant as *p* value less than 0.05 compared to the control.

Previous work has suggested that the antigrowth effect of resveratrol may be due to its action on a number of intracellular signalling molecules [6]. However, less is known about the effects of acetyl-resveratrol and polydatin on cancer cells, especially in 3D cell aggregates of ovarian cancer cells. We chose the SKOV-3 cell line because it has high expression of the oncogenic EGFR and Her-2 proteins, possible potential targets of the compounds. Resveratrol at 50 and 100 *μ*M was found to reduce the levels of pHer-2 (Figures 4(a) and 4(b)(A)) and pEGFR (Figures 4(a) and 4(b)(C)). There was no marked reduction of pHer-2 and pEGFR at 5 and 10 *μ*M (Figures 4(b)(A) and 4(b)(C)). Higher doses of resveratrol reduced the total expression of Erk1/2 and showed a tendency to suppress pErk1/2 (Figures 4(b)(F) and 4(b)(E)).

**Figure 5:**
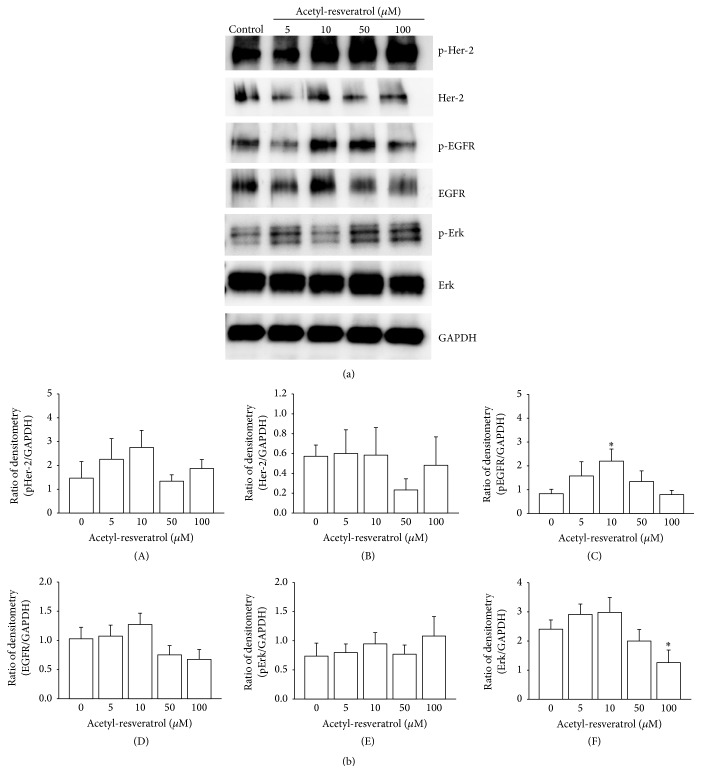
In SKOV-3 cell aggregates, acetyl-resveratrol did not alter the expression of Her-2, but it increased the phosphorylation of EGFR at 10 *μ*M (C). The total expression of Erk was significantly reduced (F). *∗* refers to statistically significant as *p* value less than 0.05 compared to the control.

**Figure 6:**
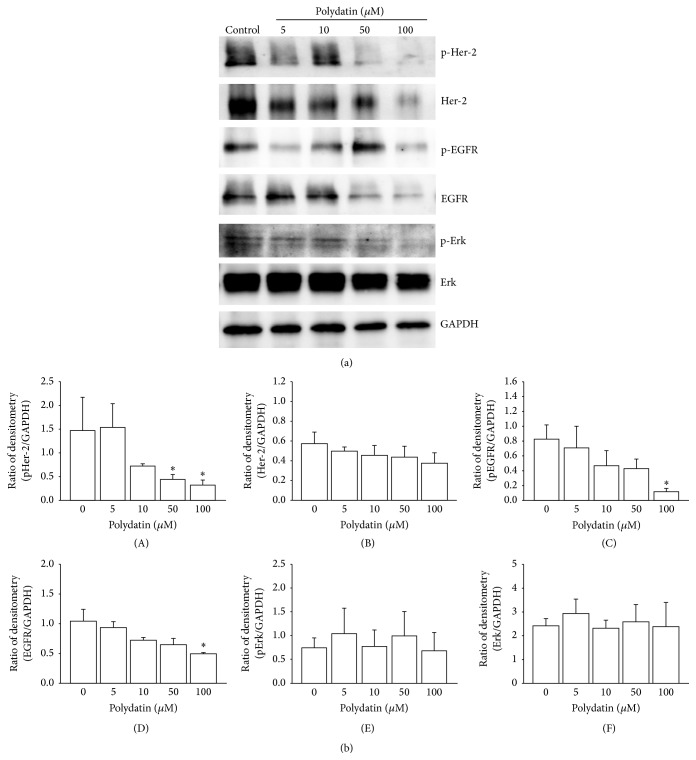
In SKOV-3 cell aggregates, the phosphorylation of Her-2 (A) and EGFR (B) was significantly reduced at higher concentrations of polydatin. The total expression of EGFR was significantly reduced with 100 *μ*M polydatin (D). There was no effect on Erk1/2 (E). *∗* refers to statistically significant as *p* value less than 0.05 compared to the control.

In contrast to resveratrol, acetyl-resveratrol did not reduce pHer-2 and pEGFR at the higher concentrations. There was an increase in pHer-2 (Figure 5(b)(A)) at 5 and 10 *μ*M and pEGFR at 10 *μ*M (Figure 5(b)(C)). Acetyl-resveratrol did not modulate the active form of Erk at any of the tested concentrations (Figure 5(b)(E)); however the expression of Erk1/2 was reduced at 100 *μ*M (Figure 5(b)(F)). Polydatin at 50 and 100 *μ*M significantly reduced the phosphorylation of Her-2 (Figures 6(a) and 6(b)(A)) and showed a significant reduction of the expression and phosphorylation of EGFR at 100 *μ*M (Figure 6(b)(C)). Polydatin did not alter the expression of Erk1/2.

**Figure 7:**
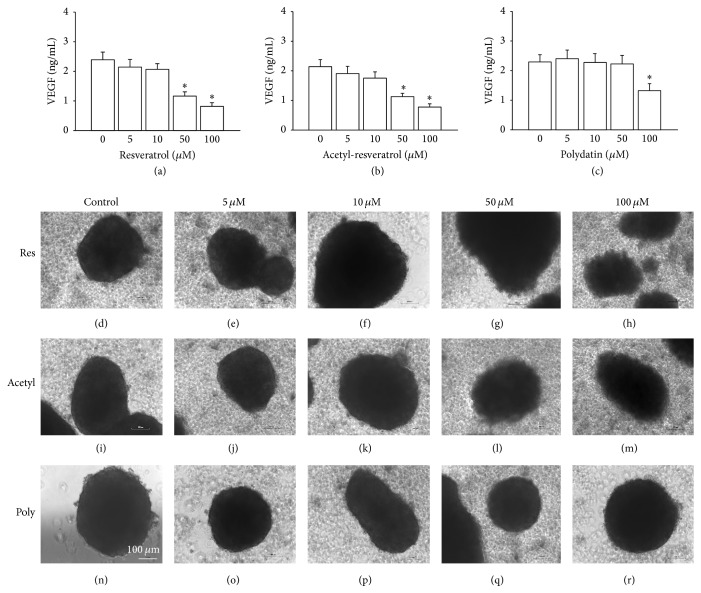
The secretion of vascular endothelial growth factor (VEGF) of SKOV-3 cell aggregates (a–c) and the morphology of OVCAR-8 3D spheroids (d–r). Higher doses of resveratrol (a), acetyl-resveratrol (b), and polydatin (c) significantly reduced the secretion of VEGF. High concentrations of resveratrol (g and h) and acetyl-resveratrol (l and m) caused the irregular rims of spheroids. Polydatin (q and r) did not show any effect. *∗* refers to statistically significant as *p* value less than 0.05 compared to the control.

There have been reports of the association of EGFR and Her-2 with increased expression of angiogenic proteins [5]. Vascular endothelial growth factor (VEGF) is a potent angiogenic peptide, and its elevated levels in women with advanced ovarian cancer are correlated with a poor prognosis [20]. To evaluate the effect of the compounds on secretion of VEGF by SKOV-3 cell aggregates, we performed an ELISA. Resveratrol (Figure 7(a)) and acetyl-resveratrol (Figure 7(b)) at 50 and 100 *μ*M were found to significantly reduce the secretion of VEGF by SKOV-3 cell aggregates. Again polydatin was less potent, significantly decreasing VEGF secretion only at a concentration of 100 *μ*M (Figure 7(c)).

**Figure 8:**
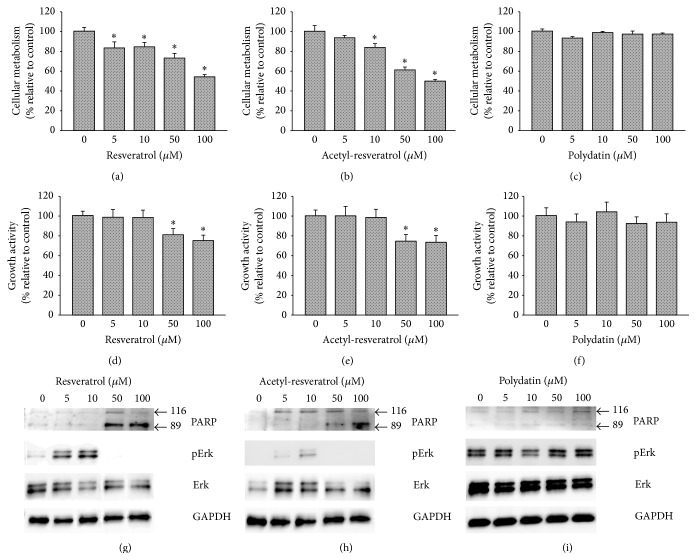
The effects of cellular metabolism, growth activity, and modulation of selective proteins following treatment of resveratrol, acetyl-resveratrol, and polydatin in OVCAR-8 spheroids. Resveratrol (a) and acetyl-resveratrol (b) reduced cellular metabolism in a dose-dependent manner. Higher concentrations of both these compounds also reduced growth activity (d and e). Polydatin showed limited effects (c and f). Both resveratrol (g) and acetyl-resveratrol (h) induced the increased expression of 89 kDa PARP-1. The activation of Erk was increased following 5 and 10 *μ* resveratrol and acetyl-resveratrol exposure. *∗* refers to statistically significant as *p* value less than 0.05 compared to the control.

To investigate the possible mechanistic basis of resveratrol and its derivatives, we also utilized the EGFR and Her-2 negative cell line, OVCAR-8. As shown in Figures 7(d)–7(r), at the higher concentrations of 50 and 100 *μ*M, resveratrol produced an irregular rim of 3D spheroids of OVCAR-8 (Figures 7(g) and 7(h)). Similar effects were observed in spheroids exposed to higher concentrations of acetyl-resveratrol (Figures 7(l) and 7(m)). At the higher concentrations, polydatin did not show any notable effect (Figures 7(q) and 7(r)). At the lower concentrations, all three compounds had no effect on spheroid morphology. Next, we measured cellular metabolism of OVCAR-8 spheroids in the presence of resveratrol and its derivatives. As shown in Figure 8, resveratrol (Figure 8(a)) and acetyl-resveratrol (Figure 8(b)) reduced cellular metabolism in a dose-dependent fashion. Polydatin did not produce this effect. We then examined the growth activity of OVCAR-8 spheroids, and higher concentrations of resveratrol (Figure 8(d)) and acetyl-resveratrol (Figure 8(e)) significantly limited growth activity whereas polydatin had no effect (Figure 8(f)). Next, we investigated the expression of EGFR, Her-2, PARP-1, Erk, and phospho-Erk. OVCAR-8 expressed very low levels of EGFR and Her-2 (data not shown). At higher concentrations, resveratrol (Figure 8(g)) and acetyl-resveratrol (Figure 8(h)) increased the level expression of 89 kDa PARP-1 suggesting that these compounds mediated the apoptotic pathway and facilitate the antigrowth activity. In contrast polydatin did not cause the increased level of PARP-1 (Figure 8(i)). There was lower level of the 89 kDa PARP-1 with the lower concentrations of three compounds. At lower concentrations of 5 and 10 *μ*M resveratrol (Figure 8(g)) and acetyl-resveratrol (Figure 8(h)) increased the activation of Erk, but higher concentrations reduced this effect. Polydatin did not modulate the activation of Erk (Figure 8(i)).

## 4. Discussion

In this study, we demonstrate antigrowth activity of resveratrol, acetyl-resveratrol, and polydatin against 3D cell aggregates of the SKOV-3 and OVCAR-8 ovarian cancer cell lines. The inhibition of growth appears to be caused by apoptosis and alteration of selective signalling proteins. The effects of these compounds were cell line dependent. In the SKOV-3 cell line, resveratrol and polydatin modulate the activation of EGFR and Her-2, but acetyl-resveratrol does not. Furthermore, secretion of the angiogenic protein, VEGF, in the SKOV-3 cell clusters is reduced in a dose-dependent fashion. In the OVCAR-8 cell line, resveratrol and polydatin modulated the activation of Erk.

Malignant cells in advanced ovarian cancer patients float in ascitic fluid as small cell 3D aggregates [3]. These cancer cell aggregates will regrow at secondary sites such as the peritoneal wall, the omentum, and the surface of internal organs. Therefore, anticancer drugs that prevent growth of aggregates and control ascites could be of great use in the treatment of advanced ovarian cancer. In the present study, we used 3D cell aggregates of the SKOV-3 and OVCAR-8 cell lines, and we have evaluated the antigrowth activity of resveratrol and the derivatives against these cell models. The SKOV-3 cell line has a high expression of the tyrosine kinase receptors, EGFR and Her-2, which have been described to be potential targets of resveratrol. OVCAR-8 cell line has low expression of these receptors, but both cell lines have distinct genetic and molecular profiles [19].

The majority of* in vitro *studies have evaluated the effects of resveratrol on cell monolayers; a cell culture model is not physiologically relevant and fails to recognize the microenvironment inside tumour tissues. In 3D cell aggregates, there is heterogeneity of cancer cells that may have differing metabolic activities due to the limitation of nutrient exposure. Furthermore, 3D cell-cell contact may play a crucial role in the autocrine activation. Therefore, this 3D model may be physiologically relevant. Consistent with previous studies, our data show that SKOV-3 and OVCAR-8 cells form large dense cell aggregates when cultured in a nonadherent condition, which mimics floating cancer cell clusters seen in the ascitic fluid of women with advanced disease [21, 22]. In the present study, we choose a range of concentrations of resveratrol and its derivatives. Concentrations can be considered physiological levels; 5 and 10 *μ*M and can be considered larger than the physiological concentrations, 50 and 100 *μ*M. Our results show that the compounds with higher concentrations lead to a decrease in the size of 3D cell aggregates, but at the more physiological concentrations they show no marked measurable effect. At the higher concentrations, the three compounds induce apoptosis in the SKOV-3 cells, but only resveratrol and acetyl-resveratrol exhibit measurable apoptosis in OVCAR-8. Our present study is in line with previous studies that the 3D cell aggregates derived from cancer cells show some inherent apoptosis and that the fraction of apoptotic cells resembles that in a solid tumour [23, 24]. It has been reported that the status of p53 is a determinant factor of apoptosis. Cancer cells with the p53 wild type are more sensitive to cytotoxic DNA damage and can undergo apoptosis more readily than p53 mutant cells [25]. However, there are also reports that apoptosis is independent of the p53 wild type or mutant [26, 27]. The SKOV-3 cell line has a null* p53* gene [28] and OVCAR-8 cell line has p53 mutation [19]. Therefore, our data is consistent with those that suggest that resveratrol and its derivatives cause apoptosis by a p53-independent pathway [29, 30].

There are a few reports suggesting effects of resveratrol on 3D cell cultures. Resveratrol has been shown to inhibit growth of 3D cell spheroids of some types of cancers [31–33]. Higher doses of resveratrol compromise the mitochondrial membrane and trigger apoptosis in spheroids of lung cancer [34]. Using the 3D cell aggregates of SKOV-3 and OVCAR-8 cells, our results confirm that at higher concentrations of resveratrol and, for the first time, acetyl-resveratrol and polydatin can alter the profile of some cell signalling molecules. We show that resveratrol and polydatin at higher concentrations can inhibit the activation of Her-2 and EGFR in SKOV-3 line and may affect growth inhibition through the attenuation of MAPK signalling cascade [35, 36]. Acetyl-resveratrol does not have the same effect. In the present study, we also used the EGFR/Her-2 negative cell line, OVCAR-8, to investigate the effect of resveratrol and its derivatives. Resveratrol and acetyl-resveratrol are able to reduce the activation of Erk following the higher concentrations, but they are likely to increase the activation of Erk at the lower concentrations suggesting that this activity may be EGFR/Her-2 independent. Bifunctional actions of resveratrol have been reported in studies of breast cancer; lower concentrations increase Her-2 expression, but higher doses showed the opposite effect [37]. We observe this effect in resveratrol and acetyl-resveratrol treated OVCAR-8 cells with the modulation of Erk activation. However, our results show that at lower concentrations acetyl-resveratrol increases the active forms of Her-2 and EGFR in SKOV-3 cells.

Differing mechanisms of action of resveratrol have been shown in different cancer cell lines using* in vitro* 2D cell monolayers. In monolayers, nonphysiological doses of resveratrol show antitumour activity via multiple signalling pathways that regulate cell growth, survival, migration, invasion, and adhesion in various types of cancer cells [6, 8, 35, 38]. There are a few reports showing that physiological concentrations of resveratrol can elicit antitumour activities, but this effect is cancer-cell-type-specific [39]. In cell monolayers of ovarian cancer cell lines, resveratrol at 25 *μ*M did not show any antiproliferative activity [40]. Mitochondrial activity was reduced after 72 hr incubation of OVCAR-3 cell line with 12.5 *μ*M resveratrol, but it did not markedly modulate signalling protein molecules [30]. Raj et al. [41] reported that the continual treatment with 0.5 and 5 *μ*M resveratrol for 6 days significantly reduced the mitochondrial activity in a cell monolayer of SKOV-3 cells but did not induce apoptosis. Our results show that resveratrol, but not acetyl-resveratrol nor polydatin, elicits antigrowth activity at lower concentrations in SKOV-3 cell aggregates, which is consistent with the previous finding in a colorectal cancer cell model [36]. Furthermore, physiological concentrations of resveratrol and acetyl-resveratrol significantly reduced cellular metabolism in OVCAR-8 spheroids.

In the current study, it is likely that the relative efficacies of resveratrol, acetyl-resveratrol, and polydatin against 3D cell aggregates may be due to their differing functional groups. Resveratrol has three hydroxyl (–OH) functional groups and by replacing these with acetyl groups (–COCH_3_) to form acetyl-resveratrol this can still elicit growth inhibition in 3D cell aggregates (Figure 1) with a similar degree of potency to resveratrol itself. However, polydatin which contains a large hydrophilic glucose molecule is less potent, and it is possible that the glucose functional group may affect the ability of polydatin to enter the cells. Our results are consistent with another study in which polydatin has been shown to have less potency than resveratrol* in vitro* [38]. Despite this, polydatin has been shown to reduce mitochondrial activity in human nasopharyngeal carcinoma cells at 5 and 10 *μ*M using the MTT assay to demonstrate the effects* in vitro* [14] which may indicate some degree of permeability and it does not inhibit DNA synthesis* in vitro* in Lewis lung carcinoma cell line at concentrations of 10 and 100 *μ*M [15].

There have been limited reports as to the mechanism of action of acetyl-resveratrol both* in vitro* and* in vivo*. Acetyl-resveratrol has been shown to inhibit the cell cycle in established cancer cell lines and have synergistic actions with cytotoxic drugs [42, 43]. At lower concentrations, it can increase the level of mRNA antioxidant proteins, suggesting that acetyl-resveratrol may act as a chemopreventive compound [12]. In a cell-free system, acetyl-resveratrol is converted back to resveratrol by the hydrolysis of the acetyl motif by intracellular esterases. Acetyl-resveratrol is metabolized and converted back to resveratrol that can be detected in plasma of a mouse model [7].

While our results show different antigrowth properties of resveratrol and its derivatives under* in vitro* conditions, their* in vivo* actions are still unknown. Therefore, it is important to confirm the data in an animal model. There are numerous studies showing that metabolized resveratrol can elicit biological activities. In one clinical trial colorectal cancer patients were given 1 gram of resveratrol daily for 8 days prior to surgical removal of the tumour. These showed a reduction of cancer cell proliferation by 5% [44]. However, after ingestion of 5 grams of resveratrol, the bioavailable level in the plasma is between 2.4 and 4.2 *μ*M. This reduces dramatically 60 minutes after ingestion [6, 11]. However, metabolized forms of resveratrol such as resveratrol-sulphate and resveratrol-glucuronide can be found at levels that are significantly higher than the parental compound in the plasma of humans [44]. Resveratrol-sulphate has been shown to inhibit NF-Kb, COX-1 and COX-2 activation in an* in vitro* study [45]. Polydatin has shown promising results in* in vivo* studies. After ingestion of polydatin, resveratrol has been found in the small intestine and faeces, suggesting that the polydatin can be converted to active resveratrol* in vivo* [13]. Polydatin has been shown to reduce the production of inflammatory molecules via the inactivation of NF-kB pathway in mice with ulcerative colitis [16]. The antitumour and antimetastatic activity of polydatin in Lewis lung carcinoma xenografts might be due to the inhibition of angiogenesis of endothelial cells [15].

VEGF is a potent angiogenic peptide secreted from a number of cells in the human body including cancer cells. Women with ovarian cancer produce high levels of VEGF and show signs of tumour progression and typically have a short-term survival [46]. Resveratrol and acetyl-resveratrol show reduction of VEGF secretion at higher concentrations, possibly associated with the reduction of cell numbers. Again, polydatin shows less of an effect. The direct targets of resveratrol with respect to its ability to suppress VEGF in cancer cells are still under investigation. Some studies suggest that resveratrol may inhibit the NF-kB pathway that may hamper the transcriptional capacity of VEGF gene promoter [6].

## 5. Conclusion

Taken together, our results suggest that resveratrol and the derivatives, acetyl-resveratrol and polydatin, have antigrowth activities against 3D cell aggregates of ovarian cancer cell lines regardless of the level expression of EGFR and Her-2. The growth inhibition is concentration dependent. The interaction of resveratrol and its derivatives, acetyl-resveratrol and polydatin, with signalling proteins may cause downstream events that lead to apoptosis and growth inhibition. Identification of the exact target molecules is a challenging yet promising area that warrants more research. We conclude that the antigrowth activities of derivatives of resveratrol warrant further studies especially using* in vivo* animal models in order to translate their antitumour effects prior to any clinical trial.
